# Systematic Review of the State of Knowledge About Açaí-Do-Amazonas (*Euterpe precatoria* Mart., Arecaceae)

**DOI:** 10.3390/plants14152439

**Published:** 2025-08-06

**Authors:** Sabrina Yasmin Nunes da Rocha, Maria Julia Ferreira, Charles R. Clement, Ricardo Lopes

**Affiliations:** 1 Post-Graduate Program in Botany at the National Institute for Amazonian Research, Av. André Araújo, 2936—Petrópolis, Manaus 69067-375, AM, Brazil; charlesr.clement@yahoo.com.br; 2 Juruá Institute, Rua Ajuricaba, 359—Aleixo, Manaus 69083-020, AM, Brazil; ferreira.julia2208@gmail.com; 3 Embrapa Western Amazon, Rodovia AM-010, Km 29, Manaus 69010-970, AM, Brazil

**Keywords:** ecology, ecosystems, genetic resources, productivity, systematics

## Abstract

*Euterpe precatoria* Mart. is an increasingly important palm for subsistence and income generation in central and western Amazonia with growing demand for its fruit pulp, which is an alternative source of açaí juice for domestic and international markets. This study synthesizes current knowledge on its systematics, ecology, fruit production in natural populations, fruit quality, uses, population management, and related areas, identifying critical research gaps. A systematic literature survey was conducted across databases including Web of Science, Scopus, Scielo, CAPES, and Embrapa. Of 1568 studies referencing *Euterpe*, 273 focused on *E. precatoria*, with 90 addressing priority themes. Genetic diversity studies suggest the *E. precatoria* may represent a complex of species. Its population abundance varies across habitats: the highest variability occurs in *terra firme*, followed by *baixios* and *várzeas*. *Várzeas* exhibit greater productivity potential, with more bunches per plant and higher fruit weight than *baixios*; no production data exist for *terra firme*. Additionally, *E. precatoria* has higher anthocyanin content than *E. oleracea*, the primary commercial açaí species. Management of natural populations and cultivation practices are essential for sustainable production; however, studies in these fields are still limited. The information is crucial to inform strategies aiming to promote the sustainable production of the species.

## 1. Introduction

In Amazonia there are some species with increasing economic importance. Among them, the *Euterpe precatoria* Mart. and *E. oleracea* Mart. palms stand out. These differ in terms of their distribution, morphology, phenology, fruit production, and fruit quality [1] and are the sources of açaí juice for national and international markets [2]. The species *E. precatoria*, popularly known as açaí-do-amazonas, açaí-da-terra-firme, açaí-da-mata, açaí-solteiro, or açaí-do-alto-amazonas, has gained increasing interest in the market due to its higher anthocyanin content [3]. It is an incipiently domesticated species, which means that the selected populations are little different from wild populations [4]. Indigenous Peoples and local communities use the species as food in the form of processed pulp, popularly called açaí wine, as well as for handicrafts, construction material, and medicinal uses [5]. In recent decades, açaí-do-pará wine (*E. oleracea*) has gone from a regional food to a national and international product [6,7]. Açaí-do-amazonas is following the same path and is being exported to almost all Brazilian states and some European countries, such as France and Switzerland [8]. As the production of *Euterpe* species has expanded, it is increasingly important to understand various aspects of their biology and management, both in natural and managed conditions and in cultivation.

The growth of the açaí market was accompanied by the expansion of extractivism (gathering from natural and managed populations) and commercial plantations, and currently cultivated production (87.3%) has a higher share of national production than the extractive system (12.7%) [9]. The State of Amazonas is the second largest producer of açaí in Brazil, with annual production estimated at approximately 45,000 tons of the fruit, coming from extractivism, from natural or managed populations [9], and more than 83,000 tons from commercial plantations [9], part of which may be *E. oleracea*, but there is still no information on the participation of each species in the state’s production. The State of Pará, the largest national producer, produced ~154,000 tons from managed systems and 1,388,000 tons from plantations, all of *E. oleracea* [9]. It is noteworthy that among non-timber forest products from extractivism in the Brazilian Amazon, açaí has the highest production value, exceeding USD 160,85 million in 2022 [9]. As the açaí market expands, it becomes important to differentiate one species from another, especially in terms of fruit quality.

The most recent taxonomic revision of *Euterpe* [1] suggests that *E. precatoria* has two varieties: var. *precatoria*, distributed in central Amazonia, and var. longavaginata, distributed in western Amazonia along the Andes from southern Peru to northern Colombia, and to the south of Central America. In cases where a species has a wide geographic distribution, such as *E. precatoria*, there may be unidentified genetic diversity, and this may pose problems in taxonomy [10]. A study using molecular markers found that the two varieties cluster in different parts of the phylogenetic tree [11], which suggests that they may be different species. Here, we concentrate on var. *precatoria* and will attempt to determine whether there could be other systematic problems.

*Euterpe precatoria* is so abundant in Amazonia that it is considered hyperdominant [12] and aggregations often occur in forests called açaí groves (açaizais). The species occurs in humid areas (*baixios*—swampy areas along blackwater rivers and streams, and the high *várzea* floodplains of whitewater rivers) and on *terra firme* (land that does not flood) [1]. The characteristics of the soils in these environments vary considerably and influence the abundance, regeneration, production, and adaptation of populations [13,14], which suggests the existence of ecotypes that may be of interest for breeding. The physical, chemical, and biological conditions offered in these environments can cause variation in the fruit productivity of populations [14,15] and, therefore, understanding fruit productivity in the ecosystems where *E. precatoria* occurs is important.

The chemical and nutritional qualities of açaí provide some health benefits to those who consume it [16]. However, there is little information regarding *E. precatoria* with respect to the composition of the açaí juice and processed pulp. In a highly globalized and competitive market, data on food composition are important to support commercialization [17]. Chemical composition tables need to be updated and as complete as possible to provide information that truly represents the composition of the food being sold.

There is some information on management techniques, seed technology, germination, production of seedlings, but little on population or plantation management, and this is important for preparing future technological systems. Population management is the basis of the extractive system practiced for thousands of years in the region and is capable of improvement. Ideas for a new bioeconomy in Amazonia [18,19] start from this assumption and depend on the development of good management practices, such as those existing for *E. oleracea* [20], and the public policies that support them [21]. Likewise, cultivation systems require good practices, but they do not exist for *E. precatoria* either. The agricultural practices adopted by producers are based on empirical knowledge, exchange of experiences, and information obtained through technical training, but there is no standardization in management [22]. Therefore, to develop a production system, the Brazilian Agricultural Research Corporation (Embrapa) began the genetic improvement of açaí, carrying out germplasm collections and progeny tests in Acre, Amazonas, Pará, and Roraima (Project SEG 10.23.00.118.00.00—Genetic improvement of *Euterpe olerecea* and *E. precatoria* in Amazonia), as well as studies for the development of a cultivation system (Project SEG 20.22.03.015.00.00—Technologies for the rational cultivation of açaí-do-amazonas (*Euterpe precatoria*) in western Amazonia). Given this context, this review concentrates on *E. precatoria* var. *precatoria*.

This systematic review gathers available information about systematics, ecology, fruit productivity, fruit quality, population management, cultivation, and related topics and identifies important gaps that need to be filled to contribute to the expansion of the economic importance of *E. precatoria* in central and western Amazonia.

## 2. Results

### 2.1. Botanical Classification

The Arecaceae family currently consists of 252 genera and approximately 2600 species [23]. In Brazil, there are 37 genera and around 300 species [24]. In Amazonia, six genera stand out for their socioeconomic importance: *Euterpe*, *Bactris*, *Astrocaryum*, *Mauritia*, *Oenocarpus*, and *Attalea* [25]. The genus *Euterpe* belongs to the subfamily Arecoidea and the tribe Euterpeae, together with the genera *Hyospathe*, *Neonicholsonia*, *Oenocarpus*, and *Prestoea*, all monophyletic [11,24]. The genus *Euterpe* includes seven species, five of them native to Brazil [26], of which four are found in Brazilian Amazonia: *E. catinga*, *E. longibracteata*, *E. oleracea*, and *E. precatoria* [1]. *Euterpe precatoria* is divided into two varieties: *longivaginata* and *precatoria* [1]. Likewise, *E. catinga* is divided into two varieties: *catinga* and *roraimae* [1].

Due to its wide distribution, *E. precatoria* may have different genetic lineages, some of which may be species, or the group forms a species complex. Pichardo-Macano et al. [11] demonstrated that the two botanical varieties (var. *precatoria* and var. *longivaginata*) are grouped in different parts of the phylogenetic tree, which suggests that they are not the same species. However, the sample used by these authors was small, one plant per taxon. Ramos et al. [27] used microsatellites to analyze the genetic diversity and population structure of *E. precatoria* var. *precatoria* in 19 locations in Brazilian Amazonia. The authors found three different genetic groups in a Bayesian analysis and eight groups in a discriminant analysis of principal components (DAPC). The upper Madeira River group, which includes samples from Porto Velho, Guajará Mirim, and Novo Mamoré, Rondônia, stands out from the others in both DAPC and Bayesian analyses and may represent a distinct lineage. Four groups obtained by DAPC are part of a Bayesian analysis group that includes populations from central-eastern Amazonas, from the middle Solimões River to Urucará in the lower Amazon River, including the lower Negro River, Amazonas; this Bayesian group may also represent a distinct lineage. The other group obtained by Bayesian analysis includes populations from three DAPC groups, one from the upper Solimões River, one from the middle-lower Madeira River, and Parintins, Amazonas; given this heterogeneity it is difficult to understand this Bayesian group.

According to Barreiro [28], preliminary molecular studies with microsatellites revealed that var. *longivaginata*, often with a cespitose habit, may be distinct from var. *precatoria*. In a Bayesian analysis of 395 *E. precatoria* plants from 58 different locations in Colombia, Ecuador, Peru, and Bolivia, three different genetic groups were found within *E. precatoria*; group 1 corresponds to var. *longivaginata*, found in the Chocó and Andes region of Colombia, Ecuador, Peru, and Bolivia, while var. *precatoria* were separated into two genetic groups; group 2 is found in the Amazon basin of Ecuador and Peru; group 3 shows a discontinuous pattern between the Amazonian regions of northwest Bolivia, southern Peru, and east-northeast Colombia.

Considering that three of the five taxa along the east–west axis of the Amazon basin have broad distributions, that the definition of *E. precatoria* with two varieties appears incorrect, and that there is important genetic structure in *E. precatoria* var. *precatoria*, the systematics of these taxa requires further study. To this end, Gabriel Damasco (pers. comm.), from the Federal University of Rio Grande do Norte, and Cristina Bacon, from the University of Gothenburg, are expanding the sampling of *Euterpe* species to better clarify the results of Barreiro [28], Pichardo-Marcano et al. [11], and Ramos et al. [27].

### 2.2. Botanical Descriptions

The species *E. precatoria* has a solitary, cylindrical, and smooth stem, gray in color, reaching 20 m in height and on average 16 cm in diameter at breast height in adult palms. Scars are found along the entire length of the stems demarcating the internodes left by the leaves that senesce and fall, spaced approximately 11 cm apart in vigorous palms. The roots are adventitious, visible at the base of the stem, and bright red in color when young. The leaves are pinnate, with an average of 14 leaves in adult palms, with a leaf sheath approximately 1.6 m long. Each leaf has 50 to 90 pairs of opposite leaflets, and the leaf is slightly or strongly pendulous horizontally [26]. The fruits are globose drupes, with stigma residue, purple-black in color when ripe, measuring approximately 1.5 cm in diameter and weighing on average 1.5 g [1,29]. The thin and succulent mesocarp surrounding a voluminous and hard endocarp contains a seed, with a tiny embryo and heterogeneous endosperm [1], although Aguiar and Mendonça [30] described the endosperm as homogeneous. The species is diploid, with 36 chromosomes and a genome size of 4.71 pg [31].

**Figure 1:**
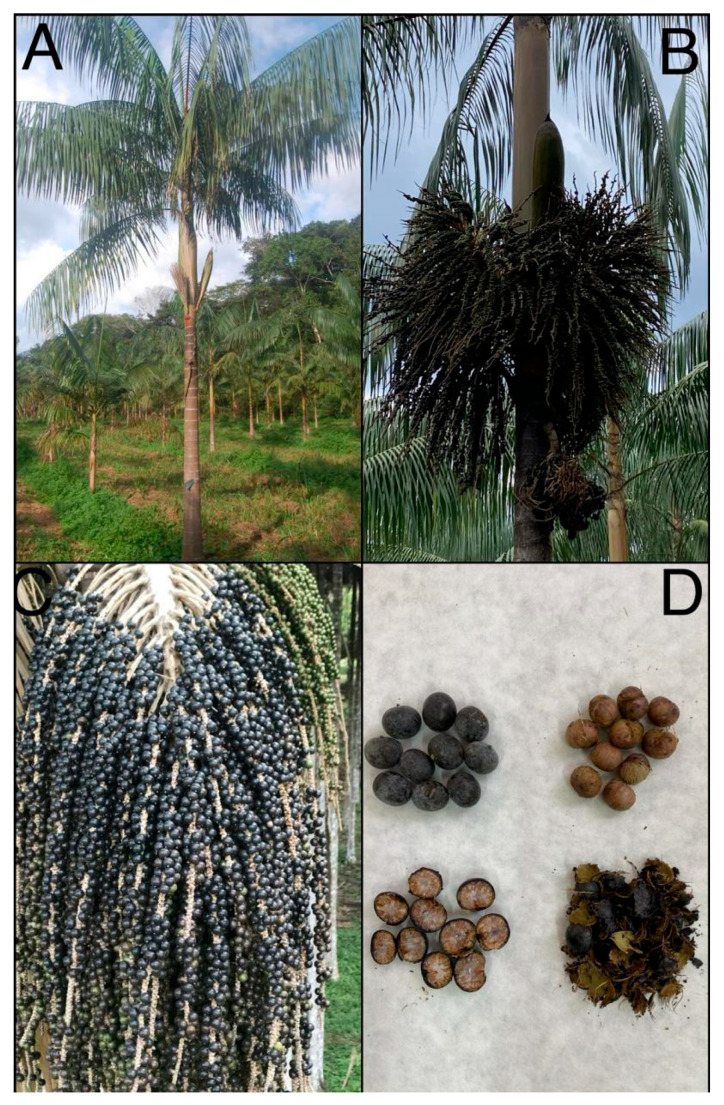
Details of the morphological characteristics of var. *precatoria*: (**A**) a young adult palm; (**B**) inflorescences and infructescences; (**C**) bunch with ripe fruits; (**D**) details of the fruits with and without pulp, cut to show endosperm, and exocarp, and mesocarp after removal.

**Figure 2:**
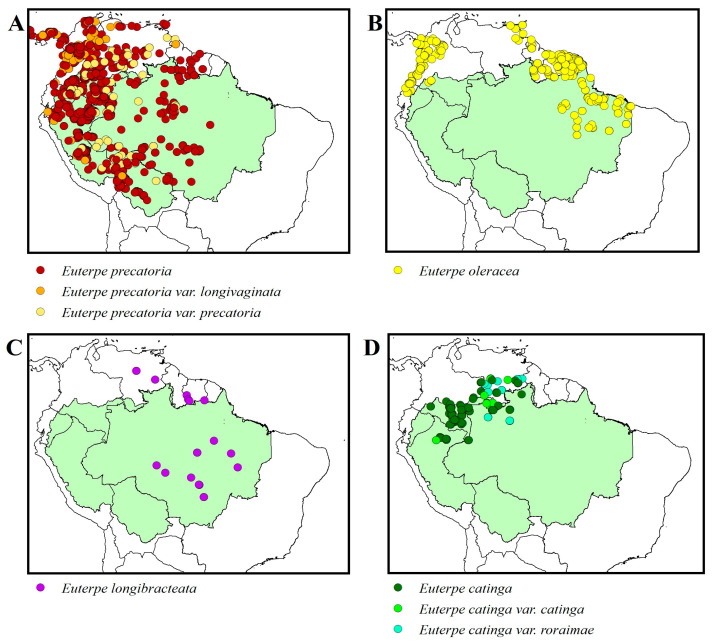
Distribution of the four *Euterpe* species and their varieties along the east–west axis of the Amazon River and northern South America: (**A**) *Euterpe precatoria*, *E. precatoria* var. *precatoria*, and *E. precatoria* var. *longivaginata*; (**B**) *E. oleracea*; (**C**) *E. longibracteata*; (**D**) *E. catinga*, *E. catinga* var. *Catinga*, and *E. catinga* var. *roraimae*. Maps in Henderson and Galeano [1], Henderson [26,33] were used to eliminate plants in GBIF that probably represent recent introductions outside of the natural and Indigenous distributions reported by these authors. Data source: GBIF.

The var. *precatoria* has grayish, solitary stems (Figure 1A); leaves with narrow leaflets, possibly pendulous, and leaf sheath that is green with yellow vertical stripes; and large inflorescences with thicker rachilla. The inflorescences are infrafoliar (Figure 1B), composed of a hard central rachis measuring 20 to 94 cm long, where an average of 200 rachillae are inserted. Adult palms produce one to four monoecious (bisexual) inflorescences per flowering period (Figure 1C), but can produce an average of 4.7 bunches per palm under good growing conditions [32]. The flowers are arranged in triads proximally, staminate in pairs or solitary distally, approximately 3.5 mm long. The fruit has a diameter of 1.0 to 1.3 cm [1] (Figure 1D). It is distributed throughout central and western Amazonia (Figure 2A). In the Bolivian Andes and in the highlands of Guyana, it is found up to 600 m in altitude [33].

The var. *longivaginata* has grayish, solitary, or rarely cespitose stems; leaves with wider leaflets that are less pendulous or horizontally arranged; smaller inflorescences with thinner rachillas; and fruits measuring 0.9 to 1.0 cm [1]. It is distributed along the eastern flank of the Andes in Peru, Ecuador, and Colombia, the extreme north of South America, Choco, and Central America (Figure 2A); in Brazil, it only occurs in the State of Acre, in the Serra do Divisor, a border region with Peru in lowland areas [24].

The species *E. longibracteata* has a solitary, occasionally cespitose stem; pinnate, divergent leaves; sheath covered externally with reddish-brown scales; infrafoliar inflorescence, branched, pendulous; and globose fruits measuring approximately 1.0 to 1.2 cm in diameter. It is distributed in Venezuela (Amazonas, Bolívar, Delta Amacuro), Guyana, and Brazil (east of Amazonas, north of Mato Grosso, west of Pará) (Figure 2C). It occurs both in floodplain forests and other humid areas, and on terra firme, always at low altitudes [1,24]. Of the species along the main axis of the Amazon basin, it is the least known.

The species *E. oleracea* has grayish cespitose stems with up to 35 stems in a clump; each stem is 3 to 20 m tall and 7 to 18 cm in diameter; pinnate, arched leaves with long-acuminate leaflets, pendulous, regularly distributed and arranged; and infrafoliar inflorescence with unisexual flowers in the same inflorescence, arranged in triads, the male ones in pairs or solitary [1]. In general, *E. oleracea* and *E. precatoria* exhibit the same morphology of reproductive organs; the inflorescences are composed of a rachis and numerous rachillas where the female and male flowers are located, ordered in triads; each female flower is flanked by two male flowers [1,34]. The fruits of *E. oleracea* are globose or ellipsoid, measuring approximately 1 to 2 cm in diameter, with a smooth epicarp, purplish-black, black, or green in color; and mesocarp with the same color as the epicarp, hard endocarp, ruminated endosperm. In Brazil, it occurs in central-eastern Pará and Amapá, forming dense populations along the rivers that form the Amazon estuary, and in Maranhão and northern Tocantins [1,33]. It also occurs in the Guiana shield to Venezuela and Colombia (Figure 2B). The species is diploid, with 36 chromosomes and a genome size of 4.22 pg [31].

The species *Euterpe catinga* has cespitose stems with few stems forming clumps, or occasionally solitary, reaching 16 m in height and 15 cm in diameter. The leaves are pinnate, with an average of 10 leaves in adult palms, with a leaf sheath approximately 1 m long, orange or reddish, green, yellowish green [1]. The fruits are globose, black-purple or reddish-brown in color with stigma residue, measuring approximately 1.3 cm in diameter; globose seeds; homogeneous endosperm [1,33].

The var. *catinga* has cespitose stems with few stems, or a solitary stem, 5 to 16 m tall and 3.5 to 9 cm in diameter. The leaves have medium leaflets with around 75 leaflets on each side. The inflorescences are infrafoliar with a central rachis 20 to 30 cm long containing around 97 rachillas [33]. The fruit has a diameter of 0.8 to 1 cm. It is distributed in western Amazonia (Figure 2D) in open forest or dwarf forests in humid and poorly drained areas on white sands in blackwater drainage areas below 350 m [1,33].

The var. *roraimae* has solitary or cespitose stems forming clumps with two to six stems, each 4 to 15 m in height and 7 to 15 cm in diameter. The leaves have medium leaflets and approximately 47 on each side, pendulous or less commonly spreading horizontally, green adaxially, lighter green abaxially. The inflorescences are infrafoliar and have a rachis 25 to 45 cm long, containing around 150 rachillas of reddish-brown or light brown color. The fruits have a diameter of 0.8 to 1.3 cm [33]. It is distributed in Venezuela, Guyana, Ecuador, and Brazil (Figure 2D) on white sand soils in humid or swampy areas in low forests, cloud forests, or dwarf forests on tepui ridges in the Guiana Highlands (or rarely on Andean slopes), at altitudes of 900 to 2100 m [1,33].

Table 1 contrasts the main botanical and agronomic characteristics of the four currently recognized *Euterpe* species and their varieties along the central Amazonian axis.

### 2.3. Phenology

Phenology is the study of plant development throughout its different stages of life: germination, emergence, growth and vegetative development, flowering, fruiting, seed formation, and maturation [35]. Knowledge of the reproductive phenology of a species is fundamental, as it identifies the periods of greatest fruit production for each species and, consequently, of the greatest availability of açaí for the market, which influences the product’s price variation. The reproductive phase of *E. precatoria* occurs annually and manifests itself from the formation of the spathe with the opening and exposure of floral buds, male floral anthesis, opening and fertilization of female flowers, development, maturation, and fruit fall [36]. In the systematic survey, seven publications deal with phenology, three of which are formal studies; one study reports information from traditional communities that exploit açaí; one study is based on information from the extension service offices of the State of Amazonas; and two are based on technical information about the açaí production in the State of Acre.

In lowland forests of the upper Urucu River in Amazonas, Peres [37] found that a majority of *E. precatoria* fruits ripen between February and July. In a comparison of the reproductive phenology of *E. precatoria* and *E. oleracea* in Manaus, Gama [38] evaluated palms for 12 months in *terra firme* forest fragments. Most *E. precatoria* inflorescence emissions occurred between August and December, and most fruit bunches were harvested between April and September, with a peak between June and July. In contrast, most *E. oleracea* inflorescence emissions occurred between February and August, and most bunches were harvested between August and December. A decade later, Lopes et al. [32] evaluated the reproductive phenology of *E. precatoria* in Manacapuru and *E. oleracea* in Rio Preto de Eva under cultivation on the *terra firme* for 24 months. They observed that 70% of *E. precatoria* inflorescence emissions occurred between July and November, and 69% of the fruit bunches were harvested between March and July. In *E. oleracea*, 79% of inflorescence emissions occurred between January and July, and 65% of the bunches were harvested between September and December. Although the harvest periods are well characterized for both species, the emission of inflorescences and harvesting of mature bunches occurred in all months of the year, except in *E. precatoria*, with no record of inflorescence emission in the month of February. The months with the lowest harvest of *E. oleracea* bunches coincide with the period with the highest harvest of *E. precatoria*.

Technicians from the extension service of the State of Amazonas observed the presence of fruits in ports and markets in all municipalities in the state [39], which represents general information about the harvest season of *E. precatoria* and *E. oleracea*. The *E. precatoria* harvest period is mainly concentrated in the first half of the year (Table 2), between the months of January and July, and differs from that observed for *E. oleracea* in Amazonas, which has its production concentrated in the months of August to November, regardless of the region of the state. The Negro–Solimões Rivers microregion includes Manaus, the state’s main market for both açaís, which explains the long period of availability.

Based on reports from residents in two locations in the Chico Mendes Extractive Reserve, in the Acre River Valley in the State of Acre, fruiting of *E. precatoria* occurs between the months of March and September [14]. An additional important observation is that there is a distinction between environments, with fruiting first in *baixios* and then in *terra firme* forests, which appears to be the norm in Acre. According to Cartaxo et al. [40], based on data collected by Sebrae in the municipality of Feijó in 2017, fruit production in *baixio* areas occurs between the months of December and July and on *terra firme* between July and December; this is different from data collected in the region of the Upper Acre River, in the municipality of Epitaciolândia, where production is concentrated in *baixio* areas from the beginning of March to the beginning of June, and in *terra firme* areas from June to October. In the Acre River Valley, in the municipality of Tarauacã, Wadt et al. [41] report that the production of *E. precatoria* in *baixio* areas occurs from January to April and in *terra firme* areas from July to September.

**Table 2:** Phenology of *Euterpe precatoria* in Amazonas, Brazil. * flowering; x fruiting.

Location or River ^3^	Jan	Feb	Mar	Apr	May	Jun	Jul	Aug	Sep	Oct	Nov	Dec
Manaus, AM ^1^	*	*	*	x	x	x	x	x	x			
Manaus, AM ^2^					x	x	*x	*	*	*	*	
Amazon River ^4^		x	x	x	x	x	x	x				
Negro–Solimões Rivers ^4^	x	x	x	x	x	x	x	x	x	x		
Madeira River ^4^	x	x	x	x	x	x					x	x
Upper Negro River ^4^	x	x	x	x							x	x
Jutaí–Juruá–Solimões Rivers ^4^	x	x	x	x	x							x
Purus River ^4^	x	x	x	x	x	x					x	
Upper Solimões River ^4^	x	x	x	x	x	x						

^1^ Gama [38]; ^2^ Lopes et al. [32]; ^3^ Seplanct, p. 19 [42]; ^4^ Melo et al. [39].

Phenological studies that indicate differences in the seasonality of fruit production were carried out both in cultivated and in natural populations; however, they were carried out in few locations, years, and populations. Studies in different pedo-climatic conditions and in different years are necessary to better understand the effects of the environment on the phenology of *E. precatoria*, especially considering climate changes that could eliminate reproduction in southern Amazonia by 2050 [43]. Considering the existing genetic variability, it is possible that different genotypes present different phenology, with earlier or later production during the year. Therefore, studies under controlled cultivation conditions with different populations and locations are necessary to better understand the effects of environment, genotype, and genotype x environment interaction on the seasonality of fruit production. The seasonality of production is of great importance for the açaí production chain, as the value of the product fluctuates greatly between the harvest period and the off season.

### 2.4. Reproductive Biology

Studies involving strategies and mechanisms related to the sexual reproduction of plants explore reproductive biology [44]. These studies identify the animals that pollinate the flowers and distribute the seeds, as well as determine the flows of pollen and seeds in the space around the palms. They also identify the mating system, the relationships between success in sexual reproduction, rates of self-fertilization and crossing, the effective number of pollen donors, and the effective size within the family, which are responsible for maintaining genetic diversity and population structure [45]. Understanding the factors that influence population structure is fundamental for improving management and conservation practices [46].

The inflorescences of *E. precatoria* are visited by many insects; the most frequent ones belong to the beetle families Staphylinidae, Chrysomelidae (*Halticinae* sp.), and Curculionidae (*Ozopherus muricatus*, *Cholus* sp., and *Phyllotrox* sp.), and bees from the Halictidae family [47,48]. In addition to these, other beetles (families Scarabaeidae, Cerambycidae, Elateridae, Brenthidae, Orthoperidae, and Dermestidae) and bees (families Apidae and Anthophoridae) visit flowers less frequently or in smaller numbers [49]. These insects have the potential to transport pollen over long distances [50], with estimates for pollen flow ranging from 50 m to 1500 m with a mean of 531 m [6,47]. Knowing the pollen flow distance is also important to define isolation distance for the establishment of open-pollinated seed production plots, because if pollen flow occurs with non-selected plants, the seeds produced will not have the expected genetic composition; thus, expected performances with the planting of improved seeds may not be achieved [47].

Most palms have zoochoric dispersion, and their fruits serve as food for birds, fruit bats, and other mammals, mainly rodents, which can act as dispersers locally [51]. As with pollen flow, zoochoric seed dispersal ranges from 4 m to 1400 m, with a mean of 400 m, with most seeds distributed in the vicinity of maternal palms, creating spatial genetic structure within populations [47]. In addition to dispersal by local animals, humans also disperse and manage both *E. oleracea* and *E. precatoria* [52].

*E. precatoria* is monoecious, with protandry (pollen is released before the female flowers are receptive), resulting in the absence of inbreeding due to the temporal separation of the anthesis of male and female flowers in the same inflorescence [53]. Ramos et al. [53] used 13 open-pollinated progenies and 13 microsatellite loci to characterize the mating system of a population of *E. precatoria* in the municipality of Parintins, Amazonas, and found a multilocus outcrossing rate of 100% (tm = 1.0), no biparental inbreeding (tm − ts = 0.001), and moderate multilocus paternity correlation (rp(m) = 0.293), and that around 30% of the progenies are formed by full siblings. Perrut-Lima et al. [54] also investigated the mating system of *E. precatoria* in three locations in different municipalities (Manacapuru, Manaquiri, and Codajás) along the lower Solimões River and detected the presence of self-fertilization, correlated mating, and biparental endogamy originating from the occurrence of spatial genetic structure within populations where open-pollinated progeny are composed of mixtures of half-sibs and full-sibs. The multilocus outcrossing rate was estimated at 1.0 (±0.01) for the Codajás population, 1.0 (±0.08) for the Manaquiri population, and 0.917 (±0.10) for the Manacapuru population, agreeing with previous reports that the species is predominantly allogamous [53]. These same authors indicate that, according to the parameters of the mating system, at least 49 reproductive palms must be sampled per population, separated by at least 100 m, for the purposes of ex situ conservation, genetic improvement and reforestation, to guarantee an effective reference population size of 150.

### 2.5. Interspecific Hybridization

The study of interspecific hybridization is important both for genetic improvement and for the in situ conservation of genetic resources. The distribution of the four *Euterpe* species in Amazonia (Figure 2) suggests that there may be several zones of natural interspecific hybridization in the region, although no hybrids have been reported to date. Interspecific hybridization allows the investigation of the possibility of combining agronomic characteristics or characteristics of interest to the market or industry present in different parental species, or if the hybrid presents hybrid vigor and outperforms the parental species for any of these characteristics [55]. The first studies on interspecific hybridization in *Euterpe* were carried out at the Campinas Agronomic Institute in the 1980s [56], but they did not include *E. precatoria*, only *E. oleracea* and *E. edulis*. Characteristics of interest in the development of açaí cultivars are average fruit weight greater than 1 g, high pulp and fruit yield, precocious production, heavy bunches, higher anthocyanin content in the pulp, tolerance to biotic (pests and diseases) and abiotic (drought) stresses, reduced height growth, and production in the off season [6,57].

Both *E. oleracea* and *E. precatoria* are allogamous, and interspecific hybridization between them is possible [58]; however, there is still little published data on the characterization and evaluation of interspecific hybrids. Lima and Oliveira [59] created two interspecific hybrid progenies, both using *E. oleracea* as a female parent and *E. precatoria* (HIE OxP) as a pollen donor, and reported on pollen viability, fruiting precocity, and fruit characteristics. However, there is no information on the fruit production potential per palm of these HIE OxP, which is necessary to estimate productivity under cultivation conditions. Delgado et al. [60] evaluated vegetative characteristics at 22 months after field planting of hybrids between *E. precatoria* x *E. oleracea* (HIE PxO) and *E. oleracea* x *E. precatoria* (HIE OxP), as well as the open-pollinated female palms. The authors reported that like *E. oleracea*, all HIE OxP plants emitted tillers, with no significant difference in the number of tillers, while in HIE PxO only 25% of the plants tillered, and in the plants that tillered, the number of tillers was lower than that of *E. oleracea* and HIE OxP. The authors also reported that HIE PxO had higher average plant height, stem diameter, and number of leaves than *E. precatoria* and no significant difference between *E. oleracea* and HIE OxP, considering only the largest stem in the clumps.

In the context of genetic resources conservation, interspecific hybridization may be important, as with the introduction of one species into the environments where the other’s natural populations occur and natural crossings and the dispersal of hybrid seeds may occur, altering the composition of these populations. Producers who collect seeds to establish *E. precatoria* plantations (there are no cultivars or commercial seeds of this species) should avoid collecting seeds from plants where there are *E. oleracea* plants nearby, as hybrid seeds may occur from natural crossing.

Considering the potential and/or possible implications of interspecific hybridization between *E. oleracea* and *E. precatoria* for genetic improvement and genetic resources conservation, more information on vegetative, reproductive, and production potential of hybrid plants under natural and cultivated conditions is important.

### 2.6. Ecological Dynamics of Euterpe precatoria in Amazonian Ecosystems

Most species in the Arecaceae family are restricted to the tropics and approximately 75% to tropical moist forests [61]. They are found in all types of soils, reliefs, and forest strata, with a variety of growth forms [62]. They are distributed in almost all environments, including dense and open forests, *terra firme*, floodplains (*várzeas*), and humid areas (*baixios*), campinas and campinaranas, performing specific functions in the structure of these ecosystems [63]. *E. oleracea* is distributed throughout the Amazon River estuary, especially in the floodplains and igapós, while *E. precatoria* is found predominantly on the *terra firme*, but also occurs along the banks of rivers with white water (*várzeas*) and black water (*igapós* and *baixios*) [33].

Under natural conditions, *E. precatoria* is adapted to nutrient-poor and well-drained environments, such as oxisols and argisols [64], and develops well in regions with annual precipitation between 1900 and 4000 mm and an average annual temperature of 26 °C. It occurs naturally in *terra firme* forests, high *várzeas*, and predominates in high densities in *baixios* [14]. It is the most abundant palm in Amazonia, with an estimated population of 5.21 billion individuals [12]. In the forest (sub-canopy) it can grow in environments with favorable or unfavorable soil, forming small aggregations of 10 to 20 palms [64] or in aggregations of 50 to 250 plants/ha [62], called açaí groves (*açaizais*). Thus, the distribution of *E. precatoria* can be considered broad, and it can be classified as common [65].

The environments where populations of *E. precatoria* occur present different conditions, demonstrating the species’ ability to adapt. *Terra firme* forests cover plateaus and hillside areas, and are areas with a more closed canopy and well-drained soils at any time of the year and are not floodable environments [14]. These forests are dominated by large trees and are characterized by a well-developed vertical structure and very high tree species diversity [66] in comparison with seasonally flooded forests [67]. Common *terra firme* soils of central Amazonia are yellow oxisols, red-yellow oxisols, and argisols, which are deep, heavily weathered, well-drained soils, with a texture varying from sandy to clayey [68]. Despite the low sum of bases, low cation exchange capacity, and low saturation index, they respond very well to fertilization, which makes the physical attributes of these soils more important than the chemical ones for cultivation [69].

The *baixios* are forests that are seasonally flooded by blackwater rivers and are directly influenced by streams (*igapós*). Their flood period can last four to five months a year, between the months of November and March [14]. These forests have a more open canopy in comparison with *terra firme* forests due to the predominance of arborescent palms and a great abundance of understory palms [70]. The soils of the *baixios* are rich in organic matter but poor in nutrients, and plinthosols, spadosols, or hydromorphic podzols and halomofic soils predominate [69].

*Várzea* environments are seasonally flooded by sediment-rich whitewater rivers; the soils are predominantly haplic gleyisols, low-humic gley or humic gley. *Várzea* soils do not have good physical properties, but they have high fertility, due to the yearly deposition of sediments, and a pH of 4.5 to 5.5 [68,69]. The *várzea* is commonly zoned by a flooding period, with the low *várzea* flooded by up to eight meters of water for a period of four to nine months per year. In contrast, the high *várzea* is not flooded annually, only in large floods. This latter type of floodplain is where *E. precatoria* occurs [71,72]. Due to the flooding regime, the *Euterpe* species adapted to these environments by developing aerial roots with lenticels and aerenchymas [69], in addition to pneumatophores that help the root system to breathe in flooded soils [73].

In data extracted from 33 publications that present the abundance per hectare of the species in the main ecosystems mentioned (*terra firme*, *baixio*, and *várzea*), the highest mean abundance is found in *terra firme* forests (Table 3). Some studies report that *baixio* forests and other humid areas are the preferred habitat for *E. precatoria*, with small aggregations close to bodies of water [14,74], or that the abundance of *E. precatoria* is greater in high *várzea* soils, followed by low *várzea* soils, but also occurs on the *terra firme* [75], which contrasts with this more complete analysis. In the *várzea* of the Putumayo River, in Colombian Amazonia, a dense population of *E. precatoria* is found, reaching 1680 individuals/ha, including approximately 248 reproductive palms [29].

The coefficients of variation of the data in Table 3 allow some inferences about the adaptation of *E. precatoria* to these environments, as the *terra firme* (CV = 81), the *baixio* (CV = 31), and the *várzea* (CV = 16) are clearly different environments. The Amazonian *terra firme* represents around 70% of the region and is composed of soils (and geological substrates) that vary greatly in texture, drainage, and nutrient availability. As a result, *terra firme* forests are very variable and offer different conditions of competition with arboreal palms, such as *E. precatoria*. The CV of abundances on *terra firme* reflects this environmental variation. *Várzeas* represent around 5% of the region [76] and have less variable and nutrient-rich soils, as well as more open forests [77], which seems to offer good conditions for *E. precatoria* and other palms. The *baixios* and other seasonally humid areas represent around 25% of the region [76] and are affected by the surrounding *terra firme*, but the soils are slightly less variable and the forests are more open, presenting suitable conditions for *E. precatoria*. Despite the different conditions offered in these environments, the main gap that needs to be filled is to carry out a more complete analysis of the species’ preferred environment.

**Table 3:** Abundance, fruit production, and productivity of *Euterpe precatoria* in three environments (*terra firme*, *baixios* (blackwater floodplain), *várzea* (whitewater floodplain)), expressed as mean ± standard deviation (minimum value–maximum value).

Environment	Abundance (Individuals/ha)	Number of Bunches (Plant/Year)	Fruit Weight (kg/Bunch)	Productivity (Tons of Fruits/ha/Year)
*Terra firme* ^1−10^	282 ± 229	-	-	-
	(69–517)	-	-	-
				
*Baixio* ^11−20^	106 ± 33	2.25 ± 0.42	5.5 ± 2.3	3
	(60–129)	(2–3)	(3–7.5)	
				
*Várzea* ^21−33^	202 ± 33	2.60 ± 0.52	8.0 ± 2.4	2.0 ± 0.3
	(170–248)	(2–3)	(5.9–11.9)	(1.8–2.2)

[78] 1; [72] 2; [79] 3; [47] 4; [80] 5; [81] 6; [82] 7; [83] 8; [84] 9; [85] 10; [14] 11; [86] 12; [87] 13; [88] 14; [89] 15; [40] 16; [74] 17; [90] 18; [15] 19; [91] 20; [92] 21; [93] 22; [29] 23; [71] 24; [94] 25; [95] 26; [96] 27; [97] 28; [98] 29; [99] 30; [100] 31; [101] 32; [26] 33.

### 2.7. Productivity

Estimates of fruit production and productivity among natural environments are important to guide the collection of genetic resources for improvement. Curiously, none of the studies on *E. precatoria* on the *terra firme* include data that allows productivity to be estimated, but there are data to compare *baixio* and *várzea* areas. Palms in the *várzea* have a greater number of fruit bunches per plant compared to the *baixios*, although the difference is not large (Table 3). An important observation is that the maximum number of fruit bunches is no more than three per palm per year, although Henderson [26] and Henderson and Galeano [1] mention a maximum of four, and an average of 4.7 (minimum 3.5; maximum 10.5) bunches was found in cultivation on the *terra firme* near Manaus [32]. Fruit weight per bunch is also higher in the *várzea*; so productivity estimates are higher than in the *baixios*. Average estimates of potential productivity can be calculated with the data in the table: potential productivity (kg/ha) = number of plants x number of bunches/plant x fruit weight/bunch. In the *várzea* this estimate is 4201 kg and in the *baixios* it is 1311 kg, which represent potential estimates that tend to be greater than measured productivity because each plant produces differently, and some do not produce every year.

In managed *várzea* areas of the Putumayo River in Colombian Amazonia, Isaza et al. [29] found an average productivity of 2.2 t/ha/year with 188 fertile palms per hectare, where each palm produces between two and four bunches/year, with a mean fruit weight per bunch of 11.6 kg. Considering the maximum number of bunches/plant/year (4), the mean weight of fruits/bunch (11.6 kg) and the number of palms (188), the estimated potential productivity is 8723 kg of fruits/ha/year.

Rocha and Viana [15] analyzed the productive potential in *baixio* areas, where they found 118 fertile palms/ha producing a mean of 2.4 bunches/palm/year and a mean of 3 kg of fruits/bunch, thus reaching an estimated productivity of 885 kg/ha/year. Phillips [102] showed that despite the low general diversity of species, flooded forests produce more fruits compared to *terra firme* forests. The existence of greater production of bunches in the *várzea* may be due to greater water availability [103], but the availability of nutrients is certainly very important, clearly resulting in greater bunch weight.

Productivity estimates for *E. precatoria* are higher in cultivation systems (monoculture or intercropping) when compared to natural forest extraction or managed forest extraction systems [90]. In addition to the greater density of individuals, in cultivation systems the arrangement of plants in the area provides less environmental competition between them, and plantings are carried out with seedlings produced with seeds obtained with some selection criterion that favors productivity. As reported by Ayres et al. [22], to obtain more productive *E. precatoria* plantations, producers in the municipality of Codajás, Amazonas, select seeds from larger bunches and smaller fruits to produce seedlings. In an *E. precatoria* cultivation system with a density of 546 plants/ha, which corresponds to a spacing of approximately 5 m × 4 m (500 plants/ha), Pinto [90] estimated productivity of 6651 t of fruits/ha.

### 2.8. Extractive Production

Investing in the production of *E. precatoria* can be strategic, since according to IBGE statistics [9] the State of Amazonas currently produces only 7.4% of the total national açaí production (extraction + cultivation), and the fact that this species occurs in the off season of *E. oleracea* certainly helps keep açaí available throughout the year [104]. Furthermore, recently the prices of *E. precatoria* products have increased due to the commercialization of processed or freeze-dried pulp as a nutritional supplement increasingly consumed in Brazil and exported to countries outside South America, mainly Europe, Canada, and the USA [105]. According to 2022 statistics from IBGE-PAM and IBGE-PEVS [9], 37.2% of the açaí production in the State of Amazonas comes from extractivism and 62.8% from cultivation. Extractivism is entirely from *E. precatoria* and, although there are no estimates of the composition of planting areas regarding the species in municipalities that stand out in açaí cultivated production in Amazonas, such as Codajás (largest producer), Coari (4th largest producer), and Anori (11th largest producer), *E. precatoria* predominates in plantations, and there are few areas cultivated with *E. oleracea*. These observations allow us to affirm that the majority of açaí production in Amazonas comes from *E. precatoria*.

The variation in açaí prices recorded in Amazonas by Conab (National Supply Company) between 2016 and 2020 [106] also corroborates this statement, as prices are lower in the period from March to July, precisely during the period when the açaí harvest is concentrated on *E. precatoria* and it is the off season of *E. oleracea*. As it is known that values are always lower during the period of greatest production, which corresponds to the period of the *E. precatoria* harvest, it can be deduced that most of the state’s production comes from this species. According to price variation records maintained by Conab, the period in which açaí has the lowest price in Amazonas coincides with the period in which açaí in Pará has the highest prices. Therefore, there is a great market opportunity to produce *E. precatoria* that can be better explored.

The extractive production of açaí in the State of Amazonas is important in Codajás, Anori, Manacapuru, Lábrea, Itacoatiara, Tapauá, Caapiranga, Borba, Tefé, and Humaitá. The municipality of Codajás stands out; its açaí groves occur around the Miuá, Badajós, Salsa, and Piorini lakes, with a production of around 26,000 tons on 488 ha of managed açaí [88]. Açaí from Codajás was recently recognized with a Geographical Indication Certification (2024). The geographical indication is considered a valuable intellectual property asset, due to the potential to add value to the product in the market [107].

In the State of Acre, *E. precatoria* is currently the main raw material used in agro-industrial production. Around 90% of all fruit pulp produced in the state is *E. precatoria* from extractivism, with the municipality of Feijó ranking first in production volume [40]. Recently, the Cooperative of Açaí Producers, Collectors, and Processors of Feijó obtained the first Geographical Indication Certification of açaí in Brazil [108].

### 2.9. Fruit Composition

Nutritional quality is one of the attributes most sought after by consumers, as açaí is considered a functional food, given its lipid, dietary fiber, and protein content [2]. Furthermore, the processed pulp of *E. oleracea* and *E. precatoria* contains approximately 90 bioactive substances, including flavonoids, phenolic compounds, lignoids, and anthocyanins, in addition to being a food relatively rich in minerals, mainly potassium, calcium, phosphorus and magnesium, vitamins E and B1, vitamin C, and carotenoids; essential fatty acids are also present in the chemical composition [7,40,109].

The chemical composition of *E. precatoria* pulp processed from different origins in central and western Amazonia identified high energy (from lipids) and mineral content, mainly potassium and calcium [3]. In the so-called functional compounds, the presence of dietary fiber from Anamã (middle Solimões River), Barcelos (middle Negro River), and Benjamin Constant (upper Solimões River) sources stands out, as do anthocyanins and oleic and linoleic fatty acids from Manaquiri (lower Solimões River), Tabatinga (upper Solimões River), and Parintins (Amazon River). From these data, it is not possible to detect geographic distribution patterns of functional quality of *E. precatoria*, which makes it difficult to determine genetic resources for quality improvement.

The great attraction for the commercialization of the açaí drink is the presence of anthocyanins, as they provide several health benefits due to their anti-free radical action, which delays aging because it prolongs the life of cells. In the immune system, they promote better blood circulation and protect the body against the accumulation of lipids in the arteries. They can delay vision loss and reduce the effects of chronic diseases. They have anticonvulsant properties and help prevent neurological diseases [16,110].

The quality of the fruit pulp of *E. precatoria* is considered superior to that of *E. oleracea* when considering antioxidant and anti-inflammatory activities [111]. According to these authors, *E. precatoria* contains strongly water-soluble antioxidants that can enter living cells and inhibit the formation of free radicals, with greater effectiveness than the antioxidants produced by *E. oleracea*. These same authors analyzed the polyphenol-rich extract from the fruit pulp that inhibited the activation of nuclear factor kappa B, suggesting that the pulp of *E. precatoria* has anti-inflammatory potential. Table 4 and Table 5 present the proximate composition based on analyses carried out in studies with processed pulp and the edible part (pulp extracted from the fruit) of *E. precatoria*, respectively.

Açaí intended for consumption as a drink, ready for consumption, or after reconstitution must comply with the characteristics established in MAPA (Ministry of Agriculture, Livestock and Food Supply Agriculture, Federal Government of Brazil) Normative Instruction No. 37 of 10/01/2018 [115]. This defines minimum identity and quality standards for açaí, clarified açaí, and dehydrated açaí, which are products obtained from the extraction with water of the edible part of the ripe fruit of both *E. oleracea* and *E. precatoria*. In the Normative Instruction, the physical, chemical, and organoleptic characteristics of each of the products derived from açaí pulp are defined. According to this government standard, there is no differentiation between the minimum identity and quality standards for the pulp of the two species. However, it is possible to envisage in the future the establishment of industry or market standards that differentiate and better value the products obtained from the pulp of *E. precatoria*, mainly due to the greater antioxidant capacity of its pulp. Geographical indications, quality certification, and traceability can contribute to the valorization of *E. precatoria* pulp.

### 2.10. Uses of Euterpe Precatoria

In Amazonia, palms are considered non-timber forest products (NTFPs) due to their uses for food, medicine, and civil construction [116,117]. Many palms have some type of utility for local communities and people in large cities as they have edible fruits and useful stems, roots, leaves, and other parts that can be used in some way, as well as being used in urban landscaping. These plant species have been meeting human needs for millennia [52]. Around 40% of Amazonian palms have economic and food value in daily life in the region [118].

Although it is important to produce palm hearts, the great commercial importance of açaí is due to the products that come from the pulp. Açaí pulp was first used to obtain a drink consumed by Indigenous Peoples, popularly known as açaí wine [119]. In Amazonia, açaí wine has been consumed as food for a long time by local populations and, after studies on its nutritional qualities [16], it began to gain new markets. As it is an energetic food, rich in anthocyanins and also contains elements such as potassium, calcium, phosphorus, magnesium, and vitamins E and B1 [3,111,120], açaí has conquered national and international markets that value its antioxidant and anti-inflammatory properties.

Açaí fruits are not consumed fresh because they have a low yield of the edible part and a relatively bland flavor. Açaí wine is a drink with a pasty consistency obtained through mechanical processing of the fruits (in pulping machines) or manually, with the addition of water during processing, which greatly facilitates pulping and filtration operations [16]. The edible fraction represents less than 30% of the total weight of açaí fruits, the remaining 70% corresponds to the seed [121]. Thus, considering the total annual fruit production of around 1.95 million tons [9], approximately 1.36 million tons of açaí seeds are generated, which require a destination more useful than disposal without generating income or other benefits. The seeds can be used in the production of cosmetics [122], to make handicrafts (bio-jewelry), or can be transformed into fertilizer; they also have a high caloric value and can be used to produce pellets intended for burning for energy production, as demonstrated for the seeds of *E. oleracea* [123]. With a little technology, furniture, acoustic panels, xaxim, and plywood, among others, can be created [7,124].

The pulp is also used for the industrial or artisanal production of ice cream, popsicles, and açaí powder and in the manufacture of jellies, sweets, cakes, coloring, and chocolates [109,125]. Other forms of product presentation are currently appearing on the market, such as pasteurized açaí, açaí mixes with guaraná syrup (*Paullinia cupana*), acerola (*Malpighia punicifolia*) and/or camu-camu (*Myrciaria dubia*), dulce de leche (milk sweet) with açaí, açaí liqueur, and isotonic drinks, among others [7,109].

The palm is also an important ornamental for use in urban landscaping, in addition to decorating home environments. Fruit production serves as food for wild and urban fauna. The leaf fibers are used to weave hats, mats, rugs, and baskets, and the stem is used to build houses and fences [126].

The species is a phytotherapeutic and medicinal alternative for communities in Amazonia who use the plant to treat illnesses. The leaves can be pressed and water added to obtain a juice used against snake bites and for muscle pain [125]. The seeds are used to prepare a dark green oil, popularly used as an antidiarrheal agent [117]. The root can be boiled, and the decoction used to treat malaria and liver and kidney infections [67,117]. From the root, the isolation of p-hydroxybenzoic acid and the lignan dihydrodiciconiferyl dibenzoate was described, the latter with marked anti-malarial activity [127]. Other products from the processing of *E. precatoria* fruits can also be economically explored. Alves et al. [128] evaluated the nutritional potential of fresh leaves, leaf flour, fresh seeds, and seed flour and showed that the peel with the pulp is a source of lipids, soluble and insoluble fibers, potassium, calcium, magnesium, and antioxidants. Fresh ground seeds are a source of insoluble fiber; sludge flour is a source of carbohydrates and insoluble fiber; and the seed and its respective flour are sources of phytic acid, condensed tannins, and antioxidants. Rufino et al. [129] reported that a bran can be obtained from the pulping residues and used to compose up to 10% of commercial laying-chicken feed. Boeira et al. [130] also highlight the feasibility of producing vinegar with attractive color and functional properties through the addition of macerated *E. precatoria* seed, creating a product that has antioxidant activity and is rich in volatile compounds. Considering the volume of seeds generated from pulp processing and that a large part of this production is not yet commercially exploited, these studies demonstrate the possibility of incorporating these co-products into food formulations, in addition to enabling efficient disposal of these residues.

### 2.11. Management of Native Populations

Management is the execution of procedures and operations that interfere with the environmental conditions of a given area to increase productivity, improve quality, and add value to the raw material [131]. In the case of native açaí populations, management focuses on two objectives: the açaí population and the populations of other species present [20,41]. This management with multiple objectives is the basis of sustainable forest management, an expanding trend worldwide [132], and can receive certification, for example from the Forest Stewardship Council. The Association of Traditional Communities of Bailique (ACTB), in Amapá, managed to certify their management plan and chain of custody for *E. oleracea* fruits in 2017 [133]. There is no similar experience with *E. precatoria* yet.

The systematic survey found only five publications that describe various aspects of the management of native açaí groves, among which are for *E. oleracea* [68] and four for *E. precatoria* [15,29,41,74]. To obtain good results, the management of native açaí groves requires organization, establishment of standards, and interest and commitment from communities. Community members who participate in management need to be trained in activities that begin with choosing the area to be managed, identifying the palms that will be harvested, and knowing how to climb the palm safely, as well as appropriate hygiene conditions for the treatment of fruits immediately after harvest.

A management plan needs to:

(i) Choose an area where there is a large concentration of the species and is easily accessible, which may be on the terra firme, in baixios, or in the high várzea [15,20,41,74];
(ii) Thin the vegetation, with the elimination of species considered to be of low commercial value [20,29];
(iii) Thin the canopy above young and adult palms to reduce mortality and increase productivity [20,29];
(iv) Enrich productive areas with selected palm seedlings [15,20,29,41];
(v) Establish harvest quotas according to the productive capacity of the population [15,20,29,41];
(vi) Select the most productive palms to use in enrichment and to have control over production estimates [41].

As açaí is a fruit that does not ripen after harvest, it is essential to collect ripe bunches, at which stage the fruit are black violet in color, with a whitish film [41]. There are several ways to harvest açaí palm bunches. The fruits can be harvested directly from the ground when the palm is young, or by climbing the palm, or even felling the palm itself. The traditional technique used by collectors is climbing, using a peconha (a piece of rope tied around the palm stem and climber’s feet). Although climbing requires effort, and picking up fallen fruit can be inefficient, both practices can guarantee the survival of the palms, as this way they do not deplete reproductive adults by felling the palm [14,29,134]. With the development of new technologies, various types of equipment are available on the market that facilitate the collection of açaí bunches [74].

Brum and Souza [72] report that during the workday identifying and climbing açaí palms in native forest, a collector spends an average of 6.5 h (from 4 am to 12 pm), collecting 1.5 to 4 bags (2.25 bags on average) and, on average, he works 52 days per harvest season (from 36 to 72 days). The commercialization unit is the bag, which weighs an average of 54.5 kg and can produce an average of 37.4 L of açaí pulp. To fill a bag, collectors must collect an average of 7.8 bunches.

After harvesting, the fruits must be removed from the rachillas and then cleaned to remove impurities (floral remains, rachillas) and placed in aerated containers, preferably shallow or guarumã (Ischnosiphon ovatus) baskets with 30 kg of fruit. As the fruits are very perishable, they must be stored in refrigerated environments, with a temperature of around 10 °C. For long-distance transport, they can be packaged in 50 kg polypropylene bags, covered with ice, or stored in cold rooms [7].

Martinot et al. [74] identified characteristics favorable for sustainable management in the açaí groves of the lower Manacapuru River and suggested adopting cultivation techniques, without completely replacing extractive management practices, as a strategy for increasing production. They also found that cultivated açaí palms grow less rapidly in height, which makes it easier to collect the fruits. This also creates a higher population density in relation to native forest populations. The authors also highlighted that the density of palms in plantations can be six to seven times greater than that observed in the forest, and collection can be concentrated in a smaller area; so there will be a significant increase in work performance with increased production.

### 2.12. Cultivation

There is no production system recommended by research for *E. precatoria*, as there already is for *E. oleracea* [20,135]. Plantings of *E. precatoria* are carried out based on empirical knowledge and exchange of experiences among producers. No cultivars are recommended by research, nor are there reports of traditional cultivars recognized by a specific name. Plantings are carried out with seedlings obtained from plants identified with good characteristics in natural populations or plantations of other producers. As the species is allogamous, the life cycle is long and the procedure for identifying individuals to be reproduced is generally based only on visual observations; significant genetic gains are not expected in new plantations [6].

When establishing a plantation, an area with suitable characteristics must be chosen, with flat or slightly undulating relief and soils with medium texture that are deep and permeable, preferably with good fertility, but it is possible to use organic and/or chemical fertilizers to improve soil fertility and provide more suitable conditions for açaí cultivation. *Euterpe precatoria* can be planted in agroforestry systems, in mixtures with other perennial or semi-perennial fruit trees or annual crops, or in monocultures [7], or even within a management plan for a native population (see 3.11). Delgado et al. [136] evaluated the morphological characterization of the growth and development of the juvenile phase of *E. precatoria* and *E. oleracea*, in response to nutritional deficiencies of boron and potassium. In *E. oleracea*, no visual symptoms of these deficiencies were shown. In *E. precatoria*, boron deficiency affects the formation of young cells and tissues, directly impacting leaf growth and the formation of reproductive organs, while potassium deficiency interferes with water regulation and photosynthesis. This may mean that for intensive cultivation, adjustments in relation to boron nutrition for *E. precatoria* should be made. However, more studies like these, on nutritional criteria and plant response to soil fertility, are needed to define fertilization recommendations with cost–benefit analysis.

The production systems of *E. precatoria* plantations in the municipality of Codajás, the largest açaí producer in Amazonas, Brazil, use spacings of 4 m x 4 m, 5 m x 4 m, and 5 m x 5 m, which are adopted based on the empirical knowledge of producers, as no publications of work evaluating the effect of spacing on development and plant production were identified in the systematic review. When managing plantations, producers use mechanical mowers once or twice a year to control spontaneous vegetation. When available on the property, producers use chicken manure as organic fertilizer (quantity not stated); most do not use soil improvers or chemical fertilizers. According to Ayres et al. [22], 38% of producers who participated in the study use chemical fertilizers, applying 300 g of NPK and 400 g of Sullfamon progressive release nitrogen fertilizer (formulas not specified) per palm/year; however, there is no information on the cost–benefit ratio of these practices.

No study on the effect of irrigation on the cultivation of *E. precatoria* was identified. There are numerous *terra firme* areas in central and western Amazonia with periods of water deficit that negatively affect production, and these areas will expand as the climate changes in the future [43]; hence irrigation may be beneficial but requires cost–benefit analyses. In *E. oleracea* plantations, irrigation has already been used in locations with three to six months of drought with positive cost–benefit results [137]. Although initially practiced empirically [7], studies on water needs have already been carried out [138], contributing to an increase in the efficient use of irrigation in the cultivation of the *E. oleracea*.

In field conditions, shadier environments promote greater leaf area, which improves the use of available light by the palm [139]. In full sun, açaí leaves have larger palisade parenchyma [140] and a higher concentration of chlorophyll per unit of leaf area, which can help against photo-destructive effects. However, high light intensity in full sunlight conditions can promote greater water loss to the environment, causing water deficit and compromising plant growth and development [141]. Almeida et al. [142] evaluated the growth of *E. precatoria* in intercropping with cooking banana cv. D’Angola in an experiment in Rio Branco, Acre, and observed that the microclimate provided by the banana canopy allowed better growth of the palms in terms of vigor and survival, a fact attributed to the lower incidence of anthracnose in shady conditions.

### 2.13. Production of Seedlings

Unlike *E. oleracea*, which in addition to reproduction through seeds can be multiplied through tillers [143], reproduction of *E. precatoria* occurs exclusively through seeds. Protocols for its in vitro micropropagation are under development [144].

Germination of *E. precatoria* seeds takes between 20 and 60 days and is classified as cryptocotyledonous, hypogeal, and adjacent ligular, as is commonly reported in genera of the Arecoideae subfamily [145,146]. Açaí seeds are recalcitrant, that is, they lose viability when dehydrated below the critical degree of humidity and, therefore, do not tolerate storage [147]. Seeds obtained from recently harvested fruits, immersed in water at room temperature for 72 h, germinate more uniformly within 14 days [148]. Seed germination can be carried out in a seedbed or directly in polyethylene bags or tubes [149].

The availability of good quality açaí seedlings is a fundamental requirement for the expansion of cultivation of the species. The use of polyethylene bags with 2.6 L of soil promotes better seedling development than smaller bags [7]. Nitrogen had a significant positive effect on the growth and quality of *E. precatoria* seedlings, while potassium had no effect [150]. Good quality *E. precatoria* seedlings were obtained after 12 months in the nursery, using 3.1 L seedling bags (18 cm in diameter and 30 cm in height) and slow-release fertilizer (8 kg m^3^ of Osmocote^®^ 15-09-12 fertilizer in the substrate) in an environment with 75% shade [142]. The use of organic substrates can also provide good quality *E. precatoria* seedlings, as reported by Araújo et al. [151] using Brazil nut shells and dried acerola seeds as substrate.

In a greenhouse study with *E. oleracea* using a yellow oxisol substrate, Viégas et al. [152] found that the demand for nutrients is presented in the order P > N > K > Mg > Mn. No similar experiment is reported for *E. precatoria* in the literature.

Several insects have already been identified attacking *E. precatoria*, including aphids, beetles, grasshoppers, whiteflies, and moths [7]. Most of them cause damage to other palms or even other fruit trees. The main fungal disease that occurs in the seedling production phase is anthracnose, caused by *Colletotrichum gloeosporioides*, which can even cause the death of seedlings, and which also affects palms in the field [153,154]. Nogueira et al. [155] reported that the chemical control of anthracnose can be carried out efficiently with the fortnightly and alternating application of the fungicides pyraclostrobin + epoxiconazole and trifloxystrobin + tebuconazole; however, these products are not registered for use with açaí. Nogueira et al. [156] used 75% shading to produce *E. precatoria* seedlings, conditions in which the seedlings showed better development and reduced anthracnose attack (<1% incidence), without the need to control the pathogen with the application of fungicides.

### 2.14. Genetic Resources

Palm heart extraction was responsible for the genetic erosion of *E. oleracea* and *E. precatoria* and the risk of extinction of *E. edulis* [157], but the new emphasis on fruit extraction is changing this situation. The conservation of selected accessions established in germplasm banks of these species is appropriate if there are genetic improvement programs, as collections allow good management conditions. The ex situ conservation of the germplasm is possible through the establishment of collections in vivo (in the field), in vitro, and in vitro with cryopreservation [158]. All the germplasm of *Euterpe* species preserved in Brazil (Table 4) is in vivo [159]. In vivo conservation facilitates the characterization and evaluation of accessions in the field, which are essential for identifying desirable genotypes for genetic improvement programs, allowing the continuous development of technologies, processes, products, and advances in knowledge. Teixeira et al. [110] affirm that the future of improvement of the genus *Euterpe* should be based on genomic selection methods with genotyping techniques of the genetic resources of the species and creation of genetic mapping allowing the selection of the most precise desirable characteristics in terms of cultivation and nutritional value.

Samples of *Euterpe* are included in germplasm banks of several Brazilian institutions in the northern region (Table 6). These banks serve as support for genetic research and mainly genetic improvement. In the Açaí Active Germplasm Bank (BAG Açaí), installed at Embrapa Amazônia Oriental, there are 307 accessions, from different origins [160]; among them is an interspecific hybrid obtained between *E. oleracea* and *E. precatoria.*

## 3. Materials and Methods

### 3.1. Literature Survey

The survey of publications for the systematic review followed the recommendations of PRISMA-EcoEvo [161], in addition to the studies by Lanza et al. [162] and Hidayati et al. [163], with appropriate modifications for the species. The survey in the systematic stage was carried out using the keywords *Euterpe precatoria* in the following online databases: Web of Science, Scopus, Scielo, CAPES (Coordination for the Improvement of Higher Education Personnel) Theses and Dissertations Catalogue, and the Embrapa Database (Appendix A—Figure A1).

The online search took place from June to August 2023 and identified 1568 publications, including 345 on *E. edulis*, 485 on *E. oleracea*, and 276 on *E. precatoria*. Titles and abstracts were examined for the first screening (Supplementary Table S1). All documents outside the scope of the study, as well as duplicate studies, were excluded from the review. The publications were then classified according to (1) title; (2) year of study; (3) type of publication, according to the classification by Lanza et al. [162], with modifications—books (n = 5), book chapters (3), articles (117), dissertations (35), and theses (17); (4) access to the database where the publication was found; and (5) research theme. Studies that addressed other *Euterpe* species without including *E. precatoria* were generally excluded.

In the second stage of the review, those studies with a primary emphasis on *E. precatoria* were considered eligible, with a total of 276 documents selected for more detailed reading. Some were excluded if (1) there was no access to the full text (8) or they were without authorized dissemination (3 dissertations), and (2) they did not address at least one of the priority issues. As a result of this screening, 90 articles were read in full. In 2024, three new studies published on *Euterpe precatoria* were included: Costa Ayres et al. [22]; Delgado et al. [136]; and Teixeira et al. [110]. In addition, Henderson [48] was included because of significant information on *Euterpe* pollination (Supplementary Table S2).

### 3.2. Quantitative Data Extraction

To compile the fruit productivity characteristics in different ecosystems, the ecosystems where the study was carried out were categorized into *terra firme* forests, *baixio* forests, and *várzea* forests. In each ecosystem, abundance per hectare and production per palm were recorded. Ideally, production per palm includes information on the number of bunches per palm, number of fruits per bunch, fruit weight per bunch (kg), bunch weight (kg), number of fruits per palm, fruit weight per palm (kg), and tons per hectare per year, but few studies were complete. When incomplete, the reported data were combined to try to complete the information, e.g., number of bunches × number of fruits per bunch = number of fruits per palm.

## 4. Conclusions

*Euterpe precatoria* is a species with increasing socioeconomic value in Amazonia, and the research being carried out could result in the generation of knowledge, products, and processes to leverage both sustainable management and cultivation of this species. The publications that address the main themes for this review (systematics, ecology, fruit productivity, nutritional quality, and management) made it possible to identify gaps in the current state of knowledge about the species that need to be addressed so that the species’ potential can be adequately explored.

The main gaps that were identified are:

*Systematics*—Determine whether *Euterpe precatoria* is more than one species or a species complex, which involves the investigation of the species’ systematics based on detailed morphological analysis, genetic information, and molecular markers and identification of divergences that may justify separating *Eutepe precatoria* into more than one species. Knowledge of systematics will contribute to more efficient strategies for conserving genetic resources and improving the species.

*Ecology*—Determine the species’ preferred environment or whether it adapts equally to all three naturally occurring environments. There may be ecotypes with specific adaptations that require identification, which involves characterization and evaluation of populations in the three different environments, including assessment of growth, flowering, and fruiting variables, as well as variables associated with fruit and pulp production in the three environments. These studies can help understand the relationship between the plant and the environment, which is necessary to guide more efficient strategies for the cultivation and management of the species, as well as for genetic improvement.

*Production of natural populations*—Identify the productive capacity of *E. precatoria* in each of these three environments, which involves investigation of the productive capacity of fruits and pulp in different natural populations in each type of environment where the species occurs. These studies will allow us to identify environments and populations with the greatest productive potential, both for the management of natural populations for production and for the selection of genotypes to compose genetic improvement populations for production in cropping systems.

*Fruit quality*—Characterize the quality of fruit pulp across the species’ distribution and in each of its three natural habitats. These studies may reveal environments that produce fruit with differentiated quality, such as higher concentrations of bioactive compounds and nutrients, which can be commercially valuable.

*Sustainable management*—Establish good management practices for each habitat where the species occurs; these provide sustainable productivity increases, ensuring long-term exploitation viability with minimal impacts on the ecosystems. Ensuring viable productivity levels is essential for sustainable management to be attractive to producers who intend to opt for a production system associated with ecosystem conservation. Production through sustainable management of natural populations can enable the acquisition of origin and quality certifications, which will allow better commercial returns, increasing producer income and encouraging environmental conservation.

*Agronomy*—Determine the most appropriate planting density, fertilization, irrigation, intercropping, and pest and disease control to achieve the species’ productivity potential in each environment. Appropriate management practices are essential for establishing healthier, more productive plantations, with reduced pest and disease incidence and improved soil conservation, which will provide a greater return and security on the producer’s investments.

*Use and conservation of genetic resources*—Conduct a broader sampling of natural populations to better quantify existing variability and understand the genetic structure of these populations, which is fundamental for strategies to conserve genetic resources and for the development of cultivars with desired agronomic characteristics, such as greater productivity, disease resistance, or adaptation to different environments.

The gaps identified are opportunities that need to be researched to make information about *E. precatoria* more comprehensive and contribute to developing its production chain, expanding its market, and generating new perspectives for its production and use.

## Figures and Tables

**Table 1:** Botanical characteristics that differentiate the four *Euterpe* species and their varieties in Amazonia [1].

Characters	*E. precatoria* var. *longivaginata*	*E. precatoria* var. *precatoria*	*E. longibracteata*	*E. oleracea*	*E. catinga* var. *catinga*	*E. catinga* var. *roraimae*
Stem	Solitary, rarely cespitose	Solitary	Solitary, occasionally cespitose	Cespitose	Cespitose with few stems, or solitary	Solitary or cespitose
Trunk diameter	4–23 cm	4–23 cm	5–8 cm	7–18 cm	3.5–9 cm	7–15 cm
Leaf	Broad leaflets, generally pendulous	Narrow leaflets, occasionally pendulous	Broad leaflets, generally pendulous	Broad leaflets, pendulous	Medium leaflets spread horizontally	Medium leaflets, pendulous
Seed	Homogeneous endosperm	Homogeneous endosperm	Homogeneous endosperm	Ruminated endosperm	Homogeneous endosperm	Homogeneous endosperm
Fruit diameter	0.9–1.0 cm	1.0–1.3 cm	1.0–1.2 cm	1.0–2.0 cm	0.8–1.0 cm	0.8–1.3
Fruit maturation	-	7–8 months	-	6 months	-	-
Number of bunches	2–4	2–4	-	2–3 per stem	-	-
Germination (time)	-	30 to 40 days	-	15 to 25 days	-	-
Eophil	Palmate	Palmate	Palmate	Bifid	Bifid	Bifid
Time to first fruiting	-	6 to 7 years	-	4 to 5 years	-	-

**Table 4:** Centesimal composition of the processed pulp of *Euterpe precatoria*. The processed pulp is açaí wine (juice) ready for consumption.

Centesimal Composition	Mean ± Standard Deviation	Minimum–Maximum
Humidity (%)	87.94 ± 3.73	84.4–94.1
Total lipids (%)	4.8 ± 2.33	1.83–9.74
Total proteins (%)	0.82 ± 0.13	0.76–1.03
Ash (%)	0.30 ± 0.08	0.2–0.46
Total fibers (%)	7.39 ± 0.32	7.1–7.15
Carbohydrates (%)	3.78	
Total titratable acidity (%)	0.29 ± 0.01	0.28–0.29
pH	4.34	-
Energy (kcal/100 g)	48.64 ± 20.05	22–91
Soluble solids (°Brix)	4.92± 2.24	3.33–6.5
Glycides (%)	0.80 ± 0.54	0.13–1.95
Concentration of macro- and microelements		
Na (mg)	2.44 ± 4.22	0.26–13.92
Ca (mg)	27.14 ± 12.05	15.99–57.85
K (mg)	122.03 ± 90.85	73.78–376.69
Fe (mg)	0.75 ± 0.25	0.46–1.16
Zn (mg)	283 ± 116.80	163.43–585.37
Bo (µg)	32.38 ± 29.11	5.17–62.24
Co (µg)	0.76 ± 0.26	0.42–1.07
Cr (µg)	58.12 ± 37.66	22.9–148.53
Functional features		
Phenolic compounds (mg GAE/100 g)	4607.4	-
Vitamin C (mg/100 g)	68.5	-
Palmitic acid (g/100 g)	1.4 ± 1.84	0.1–4.3
Oleic acid (g/100 g)	68.2 ± 5.79	58.7–74.6
Linoleic acid (g/100 g)	7.5 ± 3.64	2–11.6
Linolenic acid (g/100 g)	1.0 ± 0.40	1–1.7

Sources: Yuyama et al. [3]; Fernandes et al. [112]; Neves et al. [113].

**Table 5:** Centesimal composition of the fresh edible fruit pulp of *Euterpe precatoria*. The fresh edible pulp was extracted directly from fresh fruit with no addition of water during extraction.

Centesimal Composition	Mean ± Standard Deviation	Minimum–Maximum
Humidity (%)	30.89	-
Total lipids (%)	11.78	-
Total proteins (%)	3.31	-
Ash (%)	1.34	-
Total fibers (%)	-	-
Carbohydrates (%)	52.69	-
Functional features		
Anthocyanins (mg/100 g)	498.6 ± 226.22	128–868.9
Phenolic compounds (mg GAE/100 g)	320.7	-
Antioxidant capacity (µmol TE/g)	423.15 ± 145.45	320.3–526.0
Total carotenoids (µg/g)	963.7	-

Sources: Yuyama et al. [3]; Kang et al. [111]; Neves et al. [113]; Matos et al. [114].

**Table 6:** Existing collections in northern Brazil for ex situ conservation of the genus *Euterpe*.

Institution	Species	Accessions
Embrapa Eastern Amazon	*E. oleracea*, *E. precatoria* and *E. edulis*	307
Embrapa Amapá	*E. oleracea* and *E. precatória*	77
Embrapa Acre	*E. oleracea* and *E. precatoria*	25
Federal University of Amazonas	*E. oleracea* and *E. precatoria*	4
Federal University of Tocantins	*E. oleracea*	10
Federal University of Acre	*E. oleracea* and *E. precatoria*	5

Source: Allelo Genetic Resources Platform (Embrapa), 2023 [160] e Oliveira et al. [7].

**Figure A1:**
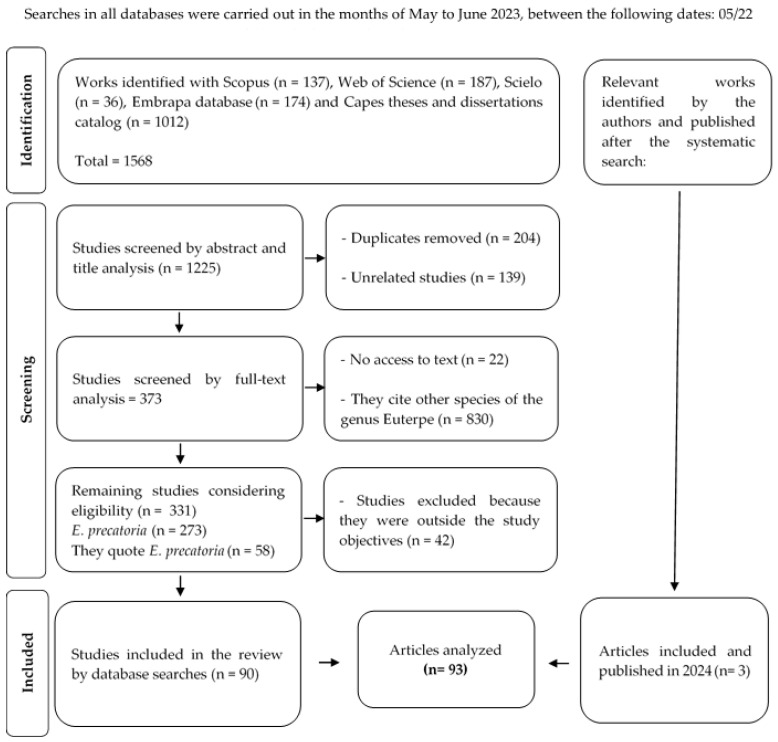
Flow diagram based on the PRISMA 2020 illustrating the phases of the process for selecting reports included in the review.

## Data Availability

All citations identified in the systematic review are presented here and in Supplementary Materials.

## Supplementary Materials

The following supporting information can be downloaded at: https://www.mdpi.com/article/10.3390/plants14152439/s1, Table S1: Spreadsheet containing the entire systematic survey of the article, Table S2: Articles analyzed based on reading of the full text.
