# Supplementary material for: Systematic Review of the State of Knowledge About Açaí-Do-Amazonas (Euterpe precatoria Mart., Arecaceae)
Source: Plants (Basel). 2025 Aug 6;14(15):2439. doi: 10.3390/plants14152439 (PMC12349038; doi:10.3390/plants14152439)
Supplement: Supplementary file 1 [file plants-14-02439-s001.zip › Supplementary Materials_Table S2_Articles analyzed based on reading of the full text.pdf]

Table S1. Articles analyzed based on reading of the full text.

Id	Authors	Title	DOI
**1**	Aguiar e Mendonça, 2003	Morfo-anatomia da semente de *Euterpe precatoria* Mart. (Palmae)	https://doi.org/10.1590/S0101-31222003000100007
**2**	Alarcón, 2005	Levantamento florístico e etnobotânico em um hectare de floresta de terra firme na região do médio Rio Negro, Roraima, Brasil	
**4**	Albuquerque et al., 2019	Densidade populacional de açaizeiros (*Euterpe precatoria* Mart.) na Região do Rio Anauá em Rorainópolis - RR	
**5**	Almeida et al., 2018	Crescimento de açaizeiro *(Euterpe precatoria* Mart.) consorciado com bananeira	
**6**	Almeida et al., 2021	Environment and slow-release fertilizer in the production of *Euterpe precatoria* seedlings	https://doi.org/10.1590/1983-40632018v4853294
**7**	Alvarez-Montalvan et al., 2021	Floristic composition, structure and tree diversity of an Amazon forest in Peru	10.17268/sci.agropecu.2021.009
**8**	Alves et al., 2022	Provenient residues from industrial processing of açaí berries (*Euterpe precatoria* Mart): Nutritional and antinutritional contents, phenolic profile, and pigments	https://doi.org/10.1590/fst.77521
**9**	Aranguren et al., 2014	Management of asaí (*Euterpe precatoria* Mart.) for fruit production in southern colombian amazonia	
**10**	Araújo et al., 2020	Use of agro-industry residues as sub strate for the production of *Euterpe precatoria* seedlings	https://doi.org/10.1590/1983-40632020v5058709
**11**	Avalos et al., 2013	Successional stage, fragmentation and exposure to extraction influence the population structure of *Euterpe precatoria* (Arecaceae)	
**12**	Costa Ayres et al., 2022	Avaliação da sustentabilidade agroecológica dos sistemas agroextrativistas do açaí-do-amazonas (*Euterpe precatoria* Martius) em Codajás	

Id	Authors	Title	DOI
**13**	Barbosa et al., 2019	Characterization of acai waste particles for civil construction use	https://doi.org/10.1590/S1517-707620190003.0750
**14**	Bayma et al., 2008	Custo e rentabilidade da atividade de extração de açaí em áreas de baixio na reserva extrativista Chico Mendes, Seringais Porvir, Filipinas, Etelvi, no Acre	
**15**	Boeira et al., 2023	Development of alcohol vinegars macerated with acai (*Euterpe precatoria* Mart.) berries and their quality evaluations with emphasis on color, antioxidant capacity, and volatiles profile	10.1111/1750-3841.16429
**16**	Brum e Souza, 2020	Flood disturbance and shade stress shape the population structure of açaí palm *Euterpe precatoria*, the most abundant amazon species	https://doi.org/10.1139/cjb-2019-0090
**17**	Brum, 2019	Ecologia e economia do açaí (*Euterpe precatoria*) em comunidades ribeirinhas na Amazônia Central	
**18**	Bussmann e Zambrana, 2012	Facing Global Markets - Usage Changes In Western Amazonian Plants: The Example Of *Euterpe Precatoria* Mart. and *E. Oleracea* Mart	
**19**	Butzke et al., 2023	Production of single assai palm, *Euterpe precatoria* Mart., seedlings subjected to nitrogen and potassium doses	https://doi.org/10.32404/rean.v10i2.7316.
**20**	Cabrera-Amaya e Rivera-Diaz, 2016	Floristic composition and structure of riparian forests of the lower basin of Pauto River, Casanare, Colombia	https://doi.org/10.15446/caldasia.v38n1.57829
**21**	Calzavara, 1972	As possibilidades do açaizeiro no estuário Amazônico	
**22**	Camargo Neves et al., 2022	Physiological maturity and wound-based orchard practices influence the antioxidant content and metabolic activity of two species of açai fruit at harvest and during storage	10.1016/j.foodchem.2022.132279
**23**	Campos et al., 2016	Rendimento de polpa de frutos de açaizeiro em áreas de baixio e terra firme em feijó, AC	

Id	Authors	Title	DOI
**24**	Carey et al, 2016	Dietary supplementation with the polyphenol-rich açaí pulps (*Euterpe oleracea* Mart. and *Euterpe precatoria* Mart.) improves cognition in aged rats and attenuates inflammatory signaling in Bv-2 microglial cells	10.1080/1028415X.2015.1115213
**25**	Cartaxo et al., 2020	*Euterpe precatoria* Mart.: Boas práticas de produção na coleta e pós-coleta de açaí-solteiro	
**26**	Castro Rodriguez et al., 2015	Asaí (*Euterpe precatoria*): Cadena de valor en el sur de le region amazónica	
**27**	Castro, 1992	Projeto de pesquisas: Extrativismo na Amazônia Central, viabilidade e desenvolvimento	
**28**	Blair e Matos et al., 2017	Economic profile of two species of genus Euterpe, producers of açaí fruits, from the Pará and Amazonas States - Brazil	10.22161/ijeab/2.4.46
**29**	Clay e Clement, 1993	Selectede species and strategies to enhance income generetio from Amazonian forest	
**30**	Cochev et al., 2019	Space-temporal dynamics of the landscape and population structure of *Euterpe precatoria* Mart. in forest fragment in the municipality of Alta Floresta, Mato Grosso State, Brazil	https://doi.org/10.5902/1980509831737
**31**	Costa, 2017	Pode o açaí (*Euterpe precatoria* Mart.) ser parte importante no desenvolvimento socioeconômico das famílias extrativistas no Acre, Brasil?	
**32**	Da Silva et al., 2015	Allometric equations for estimating biomass of *Euterpe precatoria*, the most abundant palm species in the Amazon	https://doi.org/10.3390/f6020450
**33**	De Andrade Miranda et al., 2008	Palmas de comunidades ribereñas como recurso sustentable en la Amazonía brasileña	
**34**	Díaz et al., 2014	Postharvest handling and uses of asai (*Euterpe precatoria*) fruit	10.17660/ActaHortic.2014.1047.33

Id	Authors	Title	DOI
**35**	Fernandes et al., 2016	Physicochemical composition, color and sensory acceptance of low-fat cupuaçu and açaí nectar: Characterization and changes during storage	https://doi.org/10.1590/1678-457X.03415
**36**	Ferreira et al., 2020	Morphostructural and histochemical dynamics of *Euterpe precatoria* (Arecaceae) germination	10.1007/s10265-020-01219-7.
**37**	Ferreira et al., 2022	Somatic embryogenesis and plant regeneration from zygotic embryos of the palm tree Euterpe precatoria Mart	10.1007/s11240-022-02227-2
**38**	Ferreyra-Ramos et al., 2019	Mating system analysis of açaí-do-Amazonas (*Euterpe precatoria* Mart.) using molecular markers	https://doi.org/10.1590/1984-70332019v19n1n17
**39**	Dias, 2021	Diagnóstico para o reconhecimento do açaí de Codajás Amazonas como indicação geográfica	
**40**	Gama, 2004	Estudo comparativo da biologia reprodutiva de *Euterpe oleracea* Martius e *Euterpe precatoria* Martius (Arecaceae), na região de Manaus-Am	
**41**	Gomes et al., 2016	Efeitos de gradientes ambientais na fitossociologia de assembleias de palmeiras no sudeste de Roraima, Brasil	10.31413/nativa.v4i5.3581
**42**	Henderson, 2000	The genus Euterpe in Brazil	
**43**	Isaza, et al., 2017	Demography of *Euterpe precatoria* and *Mauritia flexuosa* in the Amazon: Application of integral projection models for their harvest	10.1111/btp.12424
**44**	Jensen et al., 2002	An antiplasmodial lignan from *Euterpe precatoria*	10.1021/np020264u
**45**	Kahn e Mejia, 1990	Palm communities in wetland forest ecosystems of peruvian Amazonia	https://doi.org/10.1016/0378-1127(90)90191-D
**46**	Kahn,1988	Ecology of economically important palms in Peruvian Amazonia	

Id	Authors	Title	DOI
**47**	Kang et al., 2012	Bioactivities of açaí (*Euterpe precatoria* Mart.) fruit pulp, superior antioxidant and anti-inflammatory properties to *Euterpe oleracea* Mart	10.1016/j.foodchem.2012.01.048
**48**	Kang, J. et al., 2011	Antioxidant and anti-inflammatory activities of acai (*Euterpe precatoria*) fruit pulp	
**49**	Kuchmeister et al., 1997	Flowering, pollination, nectar standing crop, and nectaries of *Euterpe precatoria* (Arecaceae), an Amazonian rain forest palm	10.1007/BF00987942
**50**	Lima, 2012	Padrões distributivos das assembléias de palmeiras ao longo de gradiente ripário na Estação Ecológica do Cuniã, interflúvio Purus-Madeira, Rondônia	
**51**	Lopes et al., 2019	Mapping the socio-ecology of non timber forest products (Ntfp) extraction in the Brazilian Amazon: The case of acai (*Euterpe precatoria* Mart) in Acre	https://doi.org/10.1016/j.landurbplan.2018.08.025
**52**	Lopes et al., 2022	Seasonality of fruit production of *Euterpe oleracea* and *E. precatoria* cultivated in Manaus	10.18227/1982-8470ragro.v16i0.7282
**53**	Lunz, et al., 2016	Plantio de açaizeiro consorciado com bananeira: Uma alternativa de renda para o agricultor familiar	
**54**	Martinot, 2013	Manejo agro-extrativista do açaí-da-mata na Amazônia Central	
**55**	Martinot et al., 2017	Coletar ou cultivar: As escolhas dos produtores de açaí-da-mata (*Euterpe precatoria*) do Amazonas	https://doi.org/10.1590/1234-56781806-94790550408
**56**	Melo, 2022	Produtividade e sustentabilidade do cultivo de açaizeiro (*Euterpe precatoria*) no município de Humaitá – Amazonas	
**57**	Moraes, 2020	Palmeiras e usos: Espécies da Bolivia e da região	
**58**	Neves et al., 2014	Post-harvest nutraceutical behaviour during ripening and senescence of 8 highly perishable fruit species from the northern Brazilian Amazon region	10.1016/j.foodchem.2014.10.111

Id	Authors	Title	DOI
**59**	Nogueira et al., 2016	Sombreamento para controle da antracnose na produção de mudas de açaí-solteiro	
**60**	Nogueira et al., 2016	Alternativas para o controle da antracnose do açaí-solteiro	
**61**	Nogueira et al., 2016	Controle de antracnose em açaí-solteiro (Euterpe precatoria) no Acre	
**62**	Oliveira et al., 2022	*Euterpe oleracea e E. precatoria*: Açaí	
**63**		*Euterpe oleracea e E. precatoria*: Açaí	
**64**	Otarola e Avalos, 2014	Demographic variation across successional stages and their effects on the population dynamics of the neotropical palm Euterpe precatoria	
**65**	Pacheco-Palencia et al., 2019	Phytochemical composition and thermal stability of two commercial acai species, *Euterpe oleracea* and *Euterpe precatoria*	https://doi.org/10.1016/j.foodchem.2009.01.034
**66**	Pardo-Molina et al., 2020	Floristic composition of the terra firme Amazon forest of the Alto Madera sector, Bolivia	
**67**	Perrut-Lima, 2023	Genetic diversity and mating system of *Euterpe precatoria* in three localities along the lower Solimões river in Central Amazonia	10.2478/sg-2023-0008
**68**	Pichardo-Mancano, 2019	Phylogeny, historical biogeography and diversification rates In na economically important group of neotropical palms: Tribe Euterpeae	10.1016/j.ympev.2018.12.030
**69**	Pinto, 2018	Análise produtiva de sistemas agroextrativistas de açaí –da– mata (*Euterpe precatoria* Mart.) na Amazônia Central	
**70**	Ramos et al., 2021	Genetic structure in populations of *Euterpe precatoria* Mart. in the Brazilian Amazon	https://doi.org/10.3389/fevo.2020.603448
**71**	Ramos et al., 2018	Paternity analysis, pollen flow, and spatial genetic structure of a natural population of *Euterpe precatoria* in the Brazilian Amazon	https://doi.org/10.1002/ece3.4582

Id	Authors	Title	DOI
**72**	Raupp, 2010	Distribuição, abundância e fenologia reprodutiva de palmeiras em uma floresta de terra firme da Amazônia Central	
**73**	Rocha e Viana, 2004	Management of *Euterpe precatoria* Mart. (açaí) in seringal Caquetá, Acre, Brazil	
**74**	Rocha, 2004	Ecologic potential for the management of açaizeiro fruits (Euterpe precatoria Mart.) in extractive areas in Acre, Brazil	https://doi.org/10.1590/S0044-59672004000200012
**75**	Romayna et al., 2020	Evaluation of pregerminative treatments on seeds of *Euterpe precatoria* Mart. (Huasai) in the city of Pucallpa-Peru	
**76**	Rubiano et al., 1994	Physionomic, structural and floristic characterization of a forest in Sierra-Nevada de Santa-Marta, Colombia	
**77**	Rufino et al., 2020	Análise de viabilidade econômica do farelo do resíduo de açaí na alimentação de poedeiras comerciais leves	https://doi.org/10.17765/2176-9168.2020v13n3p867-882
**78**	Rufino et al., 2023	Chemical control of *Colletotrichum gloeosporioides* on seedlings of single assai palm	https://doi.org/10.32404/rean.v9i4.7264
**79**	Schauss, 2015	The effect of acai (Euterpe spp.) fruit pulp on brain health and performance	https://doi.org/10.1016/B978-0-12-411462-3.00019-9
**80**	Schulz et al., 2023	Phenolic compounds in Euterpe fruits: Composition, digestibility, and stability - A review	10.1080/87559129.2021.1909060
**81**	Shanley e Medina, 2005	Frutíferas e Plantas úteis na vida amazônica	
**82**	Smith, 2015	*Euterpe precatoria*	10.1007/978-3-319-05509-1_35
**83**	Smith et al., 2014	Açaí composition and health benefits.	
**84**	Svenning et al., 2009	Topographic and spatial controls of palm species distributions in a montane rain forest, Southern Ecuador	https://doi.org/10.1007/s10531-008-9468-3
**85**	Velarde et al., 2008	Density of adults and fruit producti on of asai (*Euterpe precatoria,* Arecaceae) in Riberalta, Bolivia	

Id	Authors	Title	DOI
**86**	Wadt et al., 2004	Manejo de açaí solteiro (*Euterpe precatoria* Mart.) para produção de frutos	
**87**	Yamaguchi et al., 2014	Amazon assai (*Euterpe precatoria*): The search for biorefinery process at the byproducts from pulp industry	10.1055/s-0034-1394934
**88**	Yepes et al., 2010	Structural recovering in andean successional forests from porce (Antioquia, Colombia)	
**89**	Yuyama et al., 2011	Caracterização físico-química do suco de açaí de *Euterpe precatoria* Mart. oriundo de diferentes ecossistemas amazônicos	https://doi.org/10.1590/S0044-59672011000400011
**90**	Zanatta, 2012	O extrativismo de açai (*Euterpe precatoria* Mart.) e os sistemas produtivos tradicionais na terra indigena Kwatá-Laranjal, Borba-Am	
**91**	Costa Ayres et al., 2024	Caracterização dos agroecossistemas de açaí-do-amazonas em Codajás, Amazonas - Brasil	10.33240/rba.v19i2.51250
**92**	Delgado et al., 2024	Morphology and allometry of juvenile açaí palms under cultivation conditions in Central Amazonia	10.3390/horticulturae10101119
**93**	Teixeira et al., 2024	A Review of the Genus *Euterpe* : Botanical and genetic aspects of açai, the purple gold of the Amazon	10.1093/botlinnean/boae060
